# Targeting polymicrobial skin infections in atopic dermatitis: role of *Pichia kudriavzevii* postbiotics as innovative tools for human health

**DOI:** 10.3389/fmicb.2026.1919460

**Published:** 2026-08-06

**Authors:** Angela Maione, Annalisa Buonanno, Lea Di Massa, Federica Carraturo, Marilena Galdiero, Eugenia Veronica Di Brizzi, Elisabetta de Alteriis, Marco Guida, Emilia Galdiero

**Affiliations:** 1 Department of Biology, University of Naples Federico II, Naples, Italy; 2 Department of Experimental Medicine, University of Campania “Luigi Vanvitelli”, Naples, Italy; 3 Dermatology Unit, University of Campania “L. Vanvitelli”, Naples, Italy

**Keywords:** biofilm, *Candida parapsilosis*, chronic wounds, inflammation, skin pathogen, *Staphylococcus aureus*

## Abstract

Atopic dermatitis (AD) is a chronic inflammatory skin disorder associated with microbial dysbiosis and persistent biofilm-forming infections, particularly involving *Candida parapsilosis* and *Staphylococcus aureus*. Postbiotics have recently emerged as promising alternatives to live probiotics due to their stability, safety, and antimicrobial properties. In this study, we evaluated the *in vitro* biological activity of two postbiotic preparations derived from the probiotic yeast *Pichia kudriavzevii* isolated from a homemade kefir: cell-free supernatant (CFS) and extracellular vesicles (EVs) against two clinical isolates of *S. aureus* and *C. parapsilosis*. While *S. aureus* demonstrated high vulnerability to postbiotic treatments, *C. parapsilosis* and the dual-species biofilm exhibited significantly higher tolerance. Results indicate a susceptibility gradient to postbiotic treatments, with *S. aureus* being the most vulnerable and *C. parapsilosis* more tolerant probably due to its structural and morphological characteristics. Treatment with CFS and EVs also reduced microbial adhesion and invasion of HaCaT keratinocytes, lowering microbial loads to 3.7–6.6 and 3.6–6.1 log_10_ CFU/mL, respectively. Furthermore, both postbiotics attenuated the inflammatory response by decreasing IL-6 and IL-8 production in pathogen-infected keratinocytes . Notably, EVs exhibited superior wound-healing activity by promoting keratinocyte migration and accelerating wound closure ( > 90% after 24 h). These findings highlight the potential of *P. kudriavzevii*-derived postbiotics as multifunctional agents capable of controlling biofilm-associated infections, modulating inflammation, and supporting tissue repair. Their application may represent a novel therapeutic approach for the management of chronic skin lesions associated with AD.

## 1 Introduction

Atopic Dermatitis (AD) is a pervasive chronic inflammatory skin disease defined by characteristic features including intense pruritus, eczematous lesions, and significant epidermal barrier dysfunction (Boguniewicz and Leung, 2011). Effective management necessitates a multi-prolonged therapeutic strategy focused not only to address immune dysregulation but also to promote healing and protection of the compromised skin barrier (Wollenberg et al., 2000).

As consequence of a compromised skin barrier and microbial dysbiosis of AD patients, the colonization by *S. aureus* is an almost universal event in AD lesions, exacerbating inflammation and impairing wound healing (Iwamoto et al., 2019). Similarly, infection by opportunistic fungi such as *C. parapsilosis* may contribute to AD pathogenesis, particularly in refractory cases (Branco et al., 2023). In addition, increasing evidence suggests that cutaneous dysbiosis involving *Candida* spp., especially *Candida albicans*, may further promote skin inflammation by disrupting microbial homeostasis and stimulating innate and adaptive immune responses through fungal cell wall components and virulence factors. Although the precise pathogenic role of *Candida*-associated dysbiosis remains to be fully elucidated, fungal overgrowth may represent an additional factor contributing to disease severity in susceptible individuals (Hülpüsch et al., 2024). The chronic nature of AD lesions often results in polymicrobial colonization. Both *S. aureus* and *C. parapsilosis* are adept at forming biofilms.

Infections associated with mixed cultures are of growing concern. In clinical settings, the co-occurrence of these pathogens can lead to the formation of mixed fungal/bacterial biofilms with enhanced resistance to conventional disinfectants and antimicrobials compared to mono-species biofilms. This represents a significant therapeutic hurdle, emphasizing the need for treatments that possess broad-spectrum efficacy against both bacterial and fungal components. Numerous studies have investigated therapeutic strategies for the management of biofilm-associated infections; however, treatment of multispecies biofilms remains highly challenging (Wang et al., 2024).

Traditional AD treatments often involve topical corticosteroids and antibiotics, which carry risks of microbial resistance and long-term side effects. Also in the context of AD, there is a growing interest for the use of probiotics which modulate health through mechanisms such as immune-stimulatory effects and inter-microorganisms competition, achieving colonization resistance against pathogens (Chae et al., 2021; Kim et al., 2024; Lee et al., 2022).

An advanced concept in microbial therapy involves postbiotics, defined as metabolic products and components secreted by beneficial probiotic microorganisms, often utilized in the form of Cell-Free Supernatant (CFS). CFS is rich in bioactive metabolites, such as organic acids, antimicrobial peptides, and signaling molecules, that can exert antimicrobial, anti-biofilm, and immunomodulatory effects. Recently, lactic acid bacteria (LAB) and the derived CFSs have been investigated for the ability to reduce the virulence of different pathogenic species, including *S. aureus* (Chae et al., 2021).

The use of CFS, rather than live bacteria, provides a therapeutic agent that is chemically defined, potentially more stable, safe and easier to standardize for topical application, enhancing its translational potential for chronic skin conditions. InAD, the continuous imbalance between harmful microbial presence and a weakened skin barrier requires a treatment capable of both reducing microbial virulence and promoting skin repair. CFS derived from probiotic species represents a promising and versatile strategy to address both aspects (Kaya et al., 2024; Li et al., 2024; Liang and Xing, 2023; Rahimi et al., 2024).

Extracellular vesicles (EVs) released by beneficial probiotic microorganisms are increasingly recognized as promising postbiotics for the prevention and management of a broad spectrum of human diseases, including infectious disorders (González-Lozano et al., 2022; Sultan et al., 2021). EVs are nanometer-scale particles produced by various cells and are composed of lipid bilayer structures containing transmembrane proteins, lipids, cytoplasmic proteins, and nucleic acids and bioactive molecules. Recently, we demonstrated that the potential probiotic yeast *Pichia kudriavzevii* isolated from a homemade kefir is capable of producing EVs that play an important role on human intestinal cells, acting as postbiotic (Maione et al., 2026).

In the present work, we have utilized two isolates of *S. aureus* and *C. parapsilosis* derived from AD patient wounds considering that the therapeutic challenge modelled *in vitro* may reflect the true virulence mechanisms and resilient phenotype encountered in the clinical management of AD (Baker et al., 2023).

Based on these premises, and aiming to explore novel alternatives to antibiotics and antifungals in the management of AD, this study was focused on evaluating:

(i) the therapeutic potential of postbiotic substances—specifically, the cell-free supernatant (CFS) and extracellular vesicles (EVs) derived from *P. kudriavzevii*, against *S. aureus*, a major contributor to the global multidrug resistance (MDR) crisis, and *C. parapsilosis*, one of the most common pathogens involved in opportunistic skin infections associated with pronounced dysbiosis.
(ii) the ability of such postbiotics to promote wound healing and reduce inflammation in immortalized human keratinocyte HaCaT cells.

## 2 Materials and methods

### 2.1 Isolation and molecular characterization of the strains

In this study, one strain of *S. aureus* and one strain of *C. parapsilosis* were used, both isolated from skin swabs collected from patients with AD presenting polymicrobial infections at Vanvitelli University Hospital using standard microbiology approach (Pastar et al., 2020). Samples were immediately transported to the laboratory under anaerobic conditions and subjected to enrichment procedures. The following day, for bacterial isolation, samples were diluted in phosphate-buffered saline (PBS) and plated on selective mannitol salt agar (Oxoid LTD., Basingstoke, Hampshire, Englands), which enables the identification of pathogenic *S. aureus* by its characteristic yellow coloration of the medium after incubation at 37 °C for 24 h. For fungal isolation, samples were inoculated onto Sabouraud Dextrose Agar (SDA; Merck, Germany) supplemented with 0.5% (*w/v*) chloramphenicol (Sigma Aldrich, Saint Louis, MO, USA) and incubated at 37 °C for 24–48 h.

Following the initial phenotypic characterization, strain identification was confirmed via molecular methods using PCR, as previously reported (Buonanno et al., 2025; Doyle, 1991). Briefly, bacterial DNA was extracted by thermal lysis using a MiniAmp™ Thermal Cycler (Thermo Fisher Scientific Inc., Waltham, MA, USA), while fungal DNA was extracted using a modified CTAB protocol. Bacterial DNA was amplified by PCR using universal primers V3_f (5′-ACT CCT ACG GGA GGC AGC AG-3′) and V6_r (5′-CGA CAG CCA TGC ANC ACC T-3′) (Cai et al., 2013), targeting a ∼700 bp region of the 16S rRNA gene. Fungal DNA amplification was carried out using the universal primer set ITS1_f (5′-GGA AGT AAA AGT CGT AAC AAG G-3′) and ITS4_r (5′-TCC TCC GCT TAT TGA TAT GC-3′) (Biofab Research, Rome, Italy), targeting the ITS-5.8S rDNA region of the fungal 18S rRNA gene (∼750 bp). PCR products were visualized on 1.5% agarose gels stained with GelRed (BIOTIUM, San Francisco, CA, USA) and compared to a 100 bp DNA ladder. The amplicons were then submitted for Sanger sequencing (Biofab Research, Rome, Italy). Resulting FASTA files were analyzed using Chromas Lite v.2.6.6 (Technelysium Pty Ltd., South Brisbane, Australia). Sequence identification was performed using BLASTN v.2.2.29 against the GenBank NCBI nucleotide database. A similarity threshold of ≥ 99% with an e-value of 0.0 was applied for molds and yeasts, and ≥ 98% with an e-value of 0.0 for most bacterial identifications (Benson et al., 2018).

### 2.2 Keratinocyte cell culture

Immortalized human keratinocyte HaCaT cells were cultured in Dulbecco’s Modified Eagle Medium (DMEM, Gibco Life Technologies) supplemented with 2 mM L- glutamine, 10% fetal bovine serum, 100 U/mL penicillin and 100 μg/mL streptomycin (Gibco Life Technologies) at 37°C with 5% CO_2_. Cells were maintained with media changes every 48 h and at approximately 60%–80% confluency.

### 2.3 Postbiotics preparation

The postbiotic cells free supernatant (CFS) derived from *P. kudriavzevii* strain isolated from a homemade kefir and previously molecular characterized (Maione et al., 2024) was used for *in vitro* experiments. As described elsewhere (Maione et al., 2025b), yeast was inoculated in YPD broth (1 % yeast extract (Sigma Aldrich, Co., St. Louis, MO, USA), 2 % Bacto Peptone (Gibco, Life Technologies Corporation, Detroit, MI, USA), 2 % glucose (SERVA Electrophoresis GmbH, Heidelberg, Germany), at 37 °C for 48 h. After incubation, the culture supernatant was collected by centrifugation at 15.000 g for 20 min, and sterile filtration was performed using a microporous membrane filter with a pore size of 0.22 μm (Corning, New York, NY, USA) to completely remove the cells. To confirm the absence of viable cells, a sterility test was performed by plating an aliquot of the filtered CFS on YPD agar plates, followed by incubation at 37 °C for 48 h. The filtered supernatant was neutralized to pH = 7.0, then lyophilized under pressure, dissolved in 2 % DMSO at final concentration of 2000 μg/mL and stored at −20°C until use. For all experiments involving CFS, DMSO vehicle controls were included at concentrations equivalent to those present in the corresponding CFS-treated conditions. Depending on the CFS concentration tested, the final DMSO concentration ranged from 0.075 to 1% (*v/v*). DMSO alone did not significantly affect the experimental parameters evaluated compared with the corresponding untreated controls. The results of the vehicle control experiments are reported in Supplementary Table 1.

The postbiotic extracellular vesicles (EVs) from *P. kudriavzevii* were prepared following a method reported in our previous study (Imparato et al., 2024). Briefly, EVs were isolated from the supernatants of *P. kudriavzevii* 48 h cultures grown in YPD medium. Supernatants were subjected to sequential centrifugation steps (4000 and 15 000 × g, 15 min, at 4°C) to remove yeasts and debris. Cell-free supernatants were then concentrated approximately 20-fold using an Amicon ultrafiltration system (cut-off, 100 kDa, Millipore) and then ultra-centrifuged (Beckman Coulter, Brea, CA, USA) at 100 000 × *g* for 1 h at 4°C. Supernatants were then discarded and EV-containing pellets washed twice with 0.1 M phosphate-buffered saline (PBS) pH 7.4 at 100 000 × *g* for 1 h at 4°C. After each isolation, 10 μL of the obtained sample containing EVs was transferred to a sterile YPD agar medium to confirm the absence of contamination with yeast cells after incubation for 48 h at 37 °C. The EV preparations had been previously examined for particle size distribution and total protein content as reported in previous study (Maione et al., 2026).

### 2.4 Minimum inhibitory concentration

The minimum inhibitory concentration (MIC) of the postbiotics against the two clinical isolates was determined using a microtiter plate assay according to Clinical and Laboratory Standards Institute guidelines (Clinical Laboratory Standards Institute, 2017, 2024). MIC assays against C. parapsilosis were performed in RPMI 1640 medium buffered with MOPS, according to guidelines, whereas MIC assays against *S. aureus* were performed in cation-adjusted Mueller–Hinton broth, according to guidelines.

Briefly, dilutions of postbiotics (CFS 75–1000 μg/mL; EVs 12.5–200 μg/mL based on protein content) and an overnight culture of each microorganism (1 × 10^3^ CFU/mL) were co-incubated in each well of the microtiter plates. Plates were incubated at 37 °C for 20 h. The cultures without postbiotics were used as a positive control, while the medium without pathogens was used as a negative control. MICs were also determined for the following antibiotics and antifungal agents: gentamicin (G) (0.5–1024 μg/mL), ampicillin (Amp) (0.5–1024 μg/mL), oxacillin (Oxa) (0.5–1024 μg/mL), amphotericin B (AmphB) (0.25–16 μg/mL), and fluconazole (FLC), ketoconazole (KETO), and itraconazole (ITRA) (0.25–64 μg/mL) to compare the antibacterial/antifungal activity.

Following the incubation period, the optical density in each well was measured at a wavelength of 590 nm using a microplate reader (Varioskan LUX Multimode Microplate Reader ThermoFisher Scientific, Waltham, MA, USA). The lowest concentration that produced ≥ 90% inhibition of microbial growth was determined as the MIC value (Preda et al., 2021).

### 2.5 Time-kill assay for co-culture of CFs/EVs and clinical isolates

Time-kill assays were performed to evaluate the temporal effects of different concentrations of CFS/EVs on the proliferation of bacterial and fungal cells. Test concentrations equivalent to sub-inhibitory (CFS 500 μg/mL, EVs 100 μg/mL) and inhibitory (CFS 1000 μg/mL, EVs 200 μg/mL) were applied to 96-well microtiter plates, each containing an initial microbial inoculum of 1 × 10^6^ CFU/mL. Untreated cells served as negative controls. Samples were incubated at 37 °C with continuous agitation. At designated time points (0, 6, 12,18, and 24 h), aliquots were collected, serially diluted, and plated onto selective media to determine viable cell counts. Microbial viability was quantified as log_10_ CFU/mL based on colony-forming units recovered from both treated and control (CTRL-) samples. All experiments were conducted in triplicate to ensure reproducibility.

### 2.6 *In vitro* biofilm assays

The ability of CFS and EVs on both to prevent biofilms formation and to eradicate a mature single and dual-species biofilms, was assessed using flat-bottom polystyrene 96-well microtiter plates, following previously established protocols (Maione et al., 2023).

Initially, the biofilm-forming capabilities of *S. aureus* C-900 and *C. parapsilosis* EN22, the two strains employed in this study, were evaluated. A standardized inoculum of 1 × 10^6^ CFU/mL (100 μL per well) was dispensed into the wells and incubated at 37 °C for 24 h. Total biofilm biomass was quantified using the crystal violet (CV) staining method (Sigma-Aldrich, Saint Louis, MO, USA). Following incubation, wells were washed three times with PBS (Oxoid, Basingstoke, UK), and biofilms were fixed at 37 °C for 1 h. A 0.2% (*v/v*) CV solution (200 μL) was added to each well and incubated at room temperature for 15 min. Excess dye was removed by washing with PBS. The retained CV was solubilized using 300 μL of 30% (*v/v*) acetic acid, and absorbance was measured at 570 nm using a microplate reader. The isolates were categorized into strongly adherent, moderately adherent, and weakly adherent (Donelli et al., 2012).

To determine the inhibitory activity against biofilm formation, increasing concentrations of CFS/EVs were added to freshly inoculated wells, followed by incubation at 37 °C for 24 h. After incubation, the biofilms were stained with CV, previously described. The percentage of biofilm inhibition was calculated using the following formula:


Biofilmformationinhibition(%)=

OD-controlOd/assayOD×control100.

To assess the ability of CFS/EVs to disrupt mature biofilms under the tested experimental conditions, the compounds were applied at various concentrations to 24-h pre-formed biofilms. The plates were then incubated for an additional 24 h at 37 °C. Subsequent staining and absorbance readings were performed as described above. The biofilm reduction was determined using the equation:


Biofilmreduction(%)=(Abs-Control

Abs)CFS/EVs/AbsControl×100

For CFUs quantification, mixed-species biofilms were treated with the highest tested concentration of CFS and EVs. After treatment, the biofilms were scraped from the bottom of the wells, suspended in PBS, and homogenized by vortexing. Serial dilutions were then prepared in PBS and plated on selective agar media to discriminate the two microbial species (YPD agar supplemented with chloramphenicol was used for *C. parapsilosis*, while Baird-Parker agar was used for *S. aureus*). Plates were incubated at 37 °C, and CFUs were counted after 24–48 h.

### 2.7 Adhesion and invasion assay

To study the inhibition of *S. aureus*/*C. parapsilosis* adhesion and invasion to HaCaT cells exerted by postbiotics CFS/EVs, cells were pre-treated with the concentrations corresponding to 500 μg/mL (CFS) and 100 μg/mL (EVs) for 2 h at 37 °C and 5% CO_2_. Afterward, microorganisms were added at a multiplicity of infection (MOI) of 10:1 and 5:1 respectively and incubated for an additional 2 h under the same conditions. The different MOIs and infection times were selected based on preliminary optimization experiments performed separately for each clinical isolate to ensure effective infection of HaCaT cells while avoiding excessive cytotoxicity and preserving monolayer integrity. The monolayers were rinsed three times with PBS, lysed by scraping and cells were recovered and diluted in PBS to be plated on corresponding selective media.

To detect invasion ability, the monolayers were incubated for an additional 2 h in DMEM supplemented with 250 μg/mL gentamicin sulfate (Sigma-Aldrich)/5 μg/mL of Amphotericin B (Alfa Aesar by Thermo Fisher Scientific, Kandel, Germany) to eliminate extracellular microorganisms. The infected monolayers were washed in PBS and then lysed with a solution of 0.1% Triton X-100 (Sigma-Aldrich, Merck, Milan, Italy) in PBS for 10 min at room temperature and processed as described above to quantify the internalized microorganisms. The number of viable microorganisms was determined in both cases by colony-forming units (CFUs/mL).

### 2.8 Cytokines ELISA assay

Cytokine quantification was performed in HaCaT cells infected with *S. aureus* or *C. parapsilosis*, either pre- or post-treatment with the most effective concentration of 500 μg/mL for CFS and 100 μg/mL for EVs. Following 24 h of incubation, cell culture supernatants were collected and centrifuged to remove debris prior to cytokine analysis. Levels of IL-6, IL-4, IL-10, IL-8, and IL-1β were quantified using commercially available ELISA kits (RayBio, Norcross GA, USA), following the manufacturer’s instructions. Absorbance was measured at 490 nm using a microplate spectrophotometer. Cytokine concentrations in the samples were calculated by comparison to standard curves generated for each cytokine.

### 2.9 Determination of postbiotics wound healing activity by scratch assay

HaCaT cells were seeded into 48-well plates and incubated for 24 h to allow the formation of a confluent monolayer. Then, a linear scratch was introduced into the monolayer using a sterile pipette tip to simulate a wound. The monolayers were subsequently infected with *S. aureus* and *C. parapsilosis* at a multiplicity of infection (MOI) of 10:1 and 5:1, respectively. The cells were then treated with CFS (500 μg/mL) or EVs (100 μg/mL) to monitor their potential in enhancing wound healing under infected conditions over time . Following 3 h of incubation, the culture medium was carefully removed, and the wells were washed with PBS. Non-infected untreated cells and infected untreated cells were included as controls.

Representative images were captured at 0, 8 and 24 h using a Nikon inverted phase contrast microscope (Nikon, Tokyo, Japan). Wound closure was quantified using the following formula:


Woundhealing(%)=[(OpenareaatT0-

OpenareaatT8or 24)/OpenareaatT0]×100

Each experimental condition was performed in quadruplicate and independently repeated three times.

### 2.10 Statistical analysis

Statistical analyses were performed using GraphPad Prism version 8.0 (GraphPad Software, San Diego, CA, USA). Data are presented as mean ± standard deviation of three independent biological replicates. One-way ANOVA followed by Tukey’s multiple-comparison test was used for experiments involving a single independent factor, whereas two-way ANOVA followed by Tukey’s multiple-comparison test was used for experiments involving two independent factors, such as treatment and concentration or treatment and time. A value of *p* < 0.05 was considered statistically significant.

## 3 Results

### 3.1 Molecular characterization of microorganisms

To evaluate, as an alternative to antibiotics/antifungals, the ability of newly identified postbiotic substances to prevent and control infected lesions in patients with AD, cultivable microorganisms present were first isolated and identified. The two clinical isolates chosen have been identified on the basis of morphology and molecular analysis, as reported in Table 1, as *S. aureus* strain C900 (Sa) and *C. parapsilosis* strain EN22 (Cp).

**TABLE 1:** Results of Sanger sequencing performed on bacterial and fungal isolates obtained from skin swabs of patients with atopic dermatitis. Preliminary identification was based on colony morphology and growth on selective media. Species identity was subsequently confirmed by PCR amplification and Sanger sequencing of the 16S rRNA gene for the bacterial isolate and the ITS1–5.8S–ITS4 region for the fungal isolate. The resulting sequences were analyzed using BLASTN (ver. 2.2.29) against the NCBI nucleotide database.

Identified microorganism	Max score	Total score	% Query cover	e-Value	% identity	Accession N.
*Staphylococcus aureus* strain C900	1230	6092	98	0.0	98.57	CP127558.1
*Candida parapsilosis* strain EN22	898	898	100	0.0	99.59	FJ809941.1

### 3.2 Antimicrobial and antibiofilm potential of the two postbiotics

First, the MIC of CFS and EVs as well as of some antibiotics and antifungals, for the two clinical isolates was calculated using the microdilution method. Results obtained are summarized in Table 2. The CFS exhibited antimicrobial activity against both tested strains, with a MIC of 1000 μg/mL against *C. parapsilosis* and *S. aureus*, whereas EVs did not inhibit the growth of both strains at 200 μg/mL. Further, both strains were susceptible to conventional antimicrobials.

**TABLE 2:** Evaluation of the minimum concentration values (MIC) of CFS and EVs inhibiting the isolates growth compared to some antifungal/antibiotics. The values were obtained from a minimum of three independent experiments.

Microorganism	CFS	EVs	AmphB	FLC	ITRA	KETO	Amp	G	Oxa
	(μg/mL)								
*C. parapsilosis*	1000	> 200	0.5	4	8	8	–	–	–
*S. aureus*	1000	> 200	–	–	–	–	0.6	1	1

AmphB, amphotericin B; FLC, fluconazole; ITRA, itraconazole; KETO, ketoconazole; Amp, ampicillin; G, gentamicin; Oxa, oxacillin; CFS, cell-free supernatant; EVs, extracellular vesicles.

**FIGURE 1:**
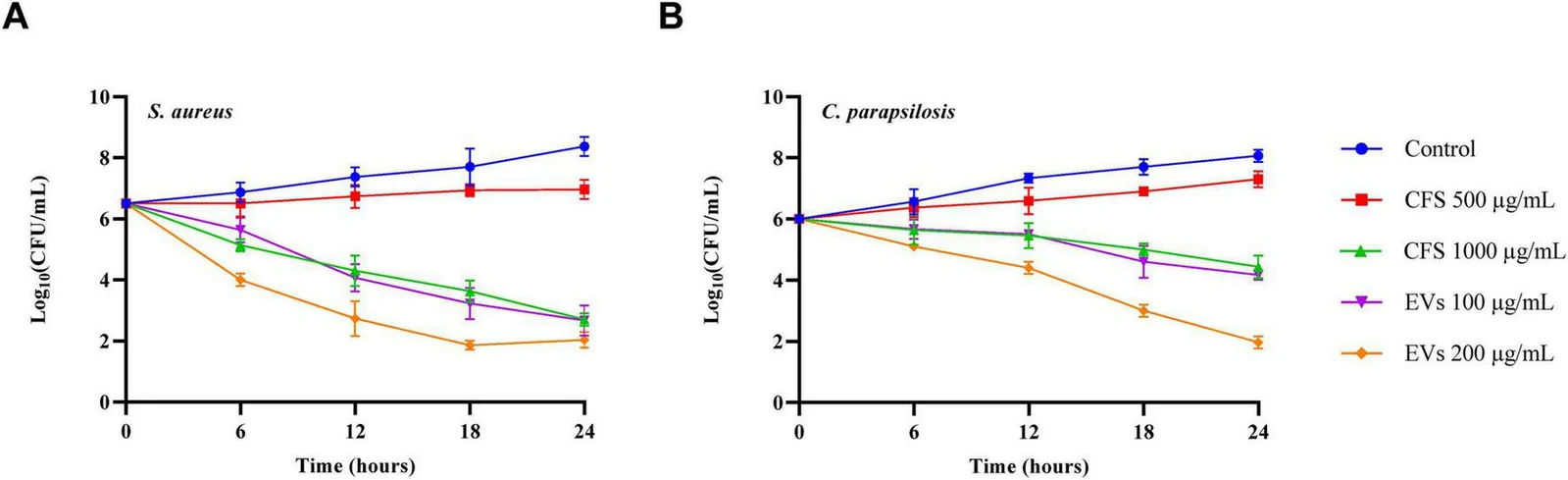
Time-kill curves of *S. aureus* **(A)** and *C. parapsilosis* **(B)** following treatment with different concentrations of CFS (500–1000 μg/mL) and EVs (100–200 μg/mL). Viable cell counts were determined at different time points and expressed as log_10_ (CFU/mL). Data are presented as mean ± SD from three independent biological experiments.

The impact of CFS and EVs on the growth of *S. aureus* and *C. parapsilosis* was evaluated by monitoring their respective growth curves over a 24-h period. As shown in Figure 1A, at concentration of 500 μg/mL, CFS slightly delayed *S. aureus* microbial growth without causing a significant reduction in viable cell numbers compared to untreated controls. At a concentration of 1000 μg/mL, CFS prevented the increase in CFU over time and induced a progressive reduction in viable cells, reaching a 5.5 log_10_ CFU/mL decrease compared with the untreated control after 24 h. Conversely, at concentration 100 μg/mL, EVs markedly impaired *S. aureus* growth, causing a progressive decrease in CFU/mL throughout the 24-h incubation period. Specifically, after 24 h, viable counts were approximately 2.7 and 2.0 log_10_ (CFU/mL) for *S. aureus* treated with EVs at 100 and 200 μg/mL, respectively, compared with about 8.3 log_10_ (CFU/mL) in the untreated control.

Figure 1B, shows the growth curve of *C. parapsilosis*. The reduction observed for *C. parapsilosis* was less pronounced than that observed for *S. aureus* under the same experimental conditions. At concentration of 500 μg/mL CFS, the decrease in growth was not significant, whereas at concentration of 1000 μg/mL a marked reduction was observed, reaching approximately 3.5 log_10_ CFU/mL. Conversely, treatment with EVs at 100 μg/mL caused a reduction of about 4 log_10_ CFU/mL, with a further decrease in viable cell numbers to around 2 log_10_ CFU/mL after 24 h when exposed to EVs at 200 μg/mL, demonstrating a clear time- and concentration-dependent inhibitory effect.

**FIGURE 2:**
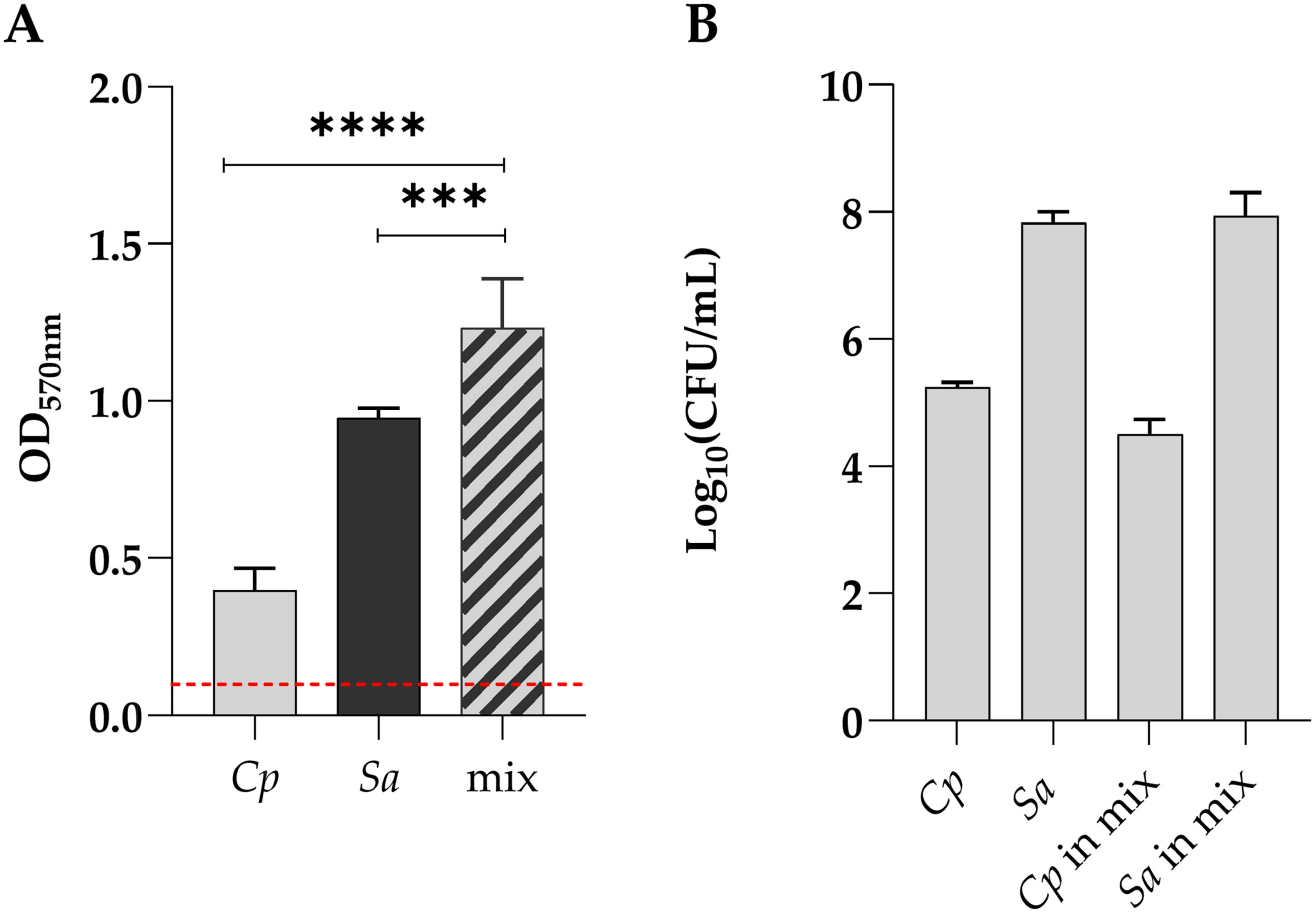
Biofilm formation by *C. parapsilosis* and *S. aureus* grown as single- and mixed-species biofilms. **(A)** Total biofilm biomass quantified by crystal violet staining and expressed as OD at 570 nm. **(B)** Viable cell counts of *C. parapsilosis* and *S. aureus* in single-species biofilms and in mixed biofilms, expressed as log_10_ (CFU/mL). *Cp*, *C. parapsilosis*; *Sa*, *S. aureus*; mix, mixed-species biofilm. Data are presented as mean ± SD from three independent biological experiments. Statistical significance was assessed by two-way ANOVA followed by Tukey’s multiple-comparison test. Only the relevant statistically significant comparisons are shown (***p = 0.001, ****p = 0.0001, Tukey’s test).

The biofilm formation assay was set up to evaluate the ability of the selected strains obtained in this study to produce biofilm. Biofilm production was performed in 96-well microtiter plates. After 24 h of incubation at 37 °C, biofilm formation was evaluated by measuring both the total biofilm cellular biomass and the cellular viability. As illustrated in Figure 2A, both isolates produced a measurable amount of biofilm after 24 h, showing a marked ability to form biofilm.Interestingly, the co-incubation of both isolates exhibited a higher biofilm-forming capacity compared to each strain tested individually. Subsequently, to determine the contribution of each species to the formation of the two types of biofilm, the colony-forming unit (CFU) counts of both *C. parapsilosis* and *S. aureus* were determined, and the counts confirmed the trend observed in the biomass data (Figure 2B). In the mixed biofilm, *C. parapsilosis* showed a slight reduction in viable cell numbers compared with its monospecific biofilm, whereas *S. aureus*-maintained levels comparable to those observed in single culture. Overall, these data indicate that *S. aureus* represents the predominant species within the mixed biofilm, while *C. parapsilosis* displays a lower viable cell burden.

**FIGURE 3:**
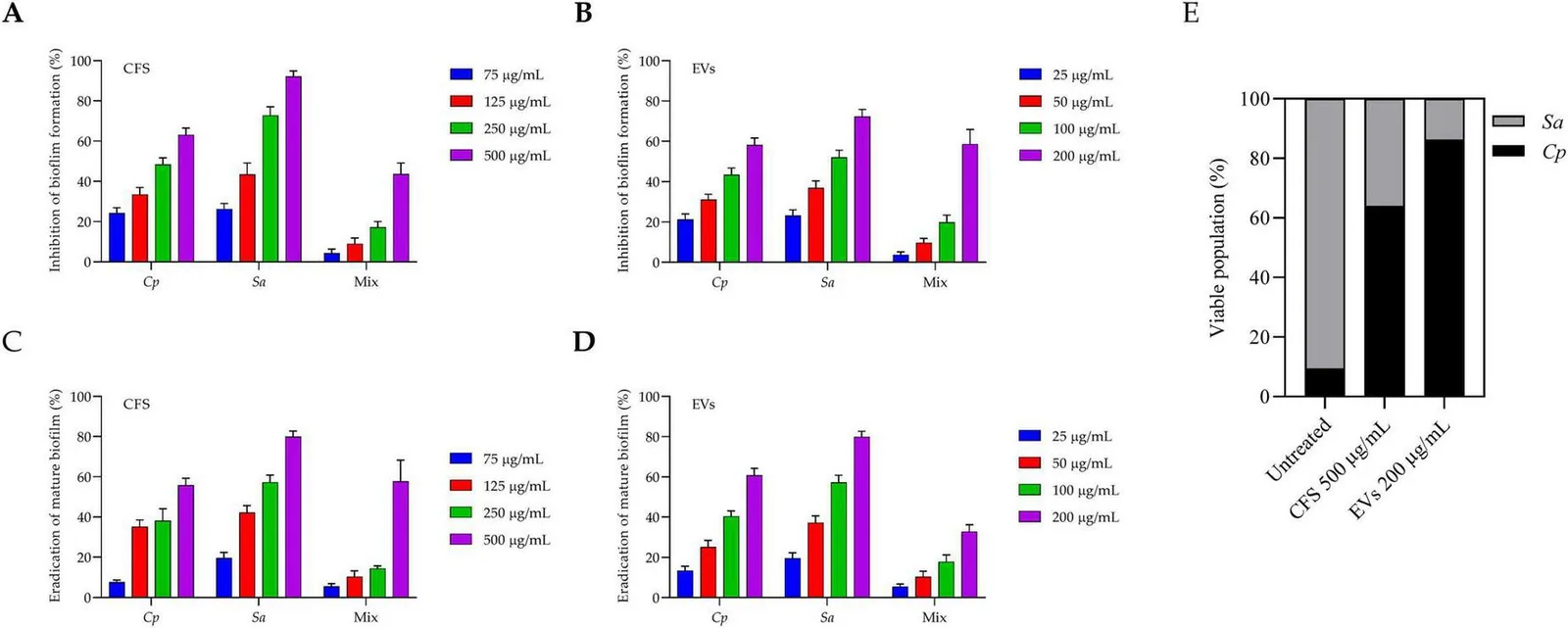
Antibiofilm activity of CFS and EVs against *C. parapsilosis* (Cp), *S. aureus* (Sa), and mixed biofilms (Mix). **(A)** Inhibition of biofilm formation by CFS at 75, 125, 250, and 500 μg/mL. **(B)** Inhibition of biofilm formation by EVs at 25, 50, 100, and 200 μg/mL. **(C)** Eradication of mature biofilm by CFS at 75, 125, 250, and 500 μg/mL. **(D)** Eradication of mature biofilm by EVs at 25, 50, 100, and 200 μg/mL. **(E)** Percentage of viable population in the mixed-species biofilm formed by *C. parapsilosis* and *S. aureus* untreated and after treatment with the highest tested concentration of the two compounds. Data are expressed as percentage of inhibition or eradication relative to untreated controls and presented as mean ± SD from three independent biological experiments.

For single- and dual-species biofilms treated with CFS and EVs, both postbiotic compounds significantly inhibited biofilm formation and reduced mature biofilms, as shown in Figure 3.

CFS was tested at concentrations from 75 to 500 μg/mL, while EVs were tested from 25 to 200 μg/mL. As shown in Figure 3A, CFS inhibited biofilm formation in a concentration-dependent manner, with the strongest effect observed at 500 μg/mL. At this concentration, biofilm inhibition reached 62 % for C. parapsilosis and 92% for *S. aureus*, whereas the mixed biofilm was less affected, showing 43% inhibition. Similarly, EVs reduced biofilm formation, with the highest concentration tested, 200 μg/mL, causing 58 % inhibition in C. parapsilosis, 72% in *S. aureus*, and 58% in the mixed biofilm (Figure 3B). Notably, EVs showed a marked effect on the mixed biofilm only at the highest concentration.

The eradication assays confirmed a similar trend against mature biofilms. CFS showed its greatest activity at 500 μg/mL, reducing mature biofilms by 60% in *C. parapsilosis*, 79% in *S. aureus*, and 55% in the mixed biofilm (Figure 3C). A comparable effect was observed for EVs, which at 200 μg/mL eradicated mature biofilms by approximately 60% in *C. parapsilosis*, 78% in *S. aureus*, and 31% in the mixed biofilm (Figure 3D).

In the untreated mixed-species biofilm, *S. aureus* represented 90% of the total viable population, whereas *C. parapsilosis* accounted for 9%. At the highest concentration tested, CFS treatment resulted in a residual mixed biofilm composed of 64% *C. parapsilosis* and 36% *S. aureus*. Furthermore, EV-treated biofilms showed a higher relative abundance of *C. parapsilosis* (86%), while *S. aureus* represented only 13% of the total viable population, indicating a marked treatment-associated shift in the relative species composition of the residual mixed biofilm (Figure 3E).

### 3.3 The two postbiotics as molecules with anti- adhesion and anti- invasion properties in mammalian cells

**FIGURE 4:**
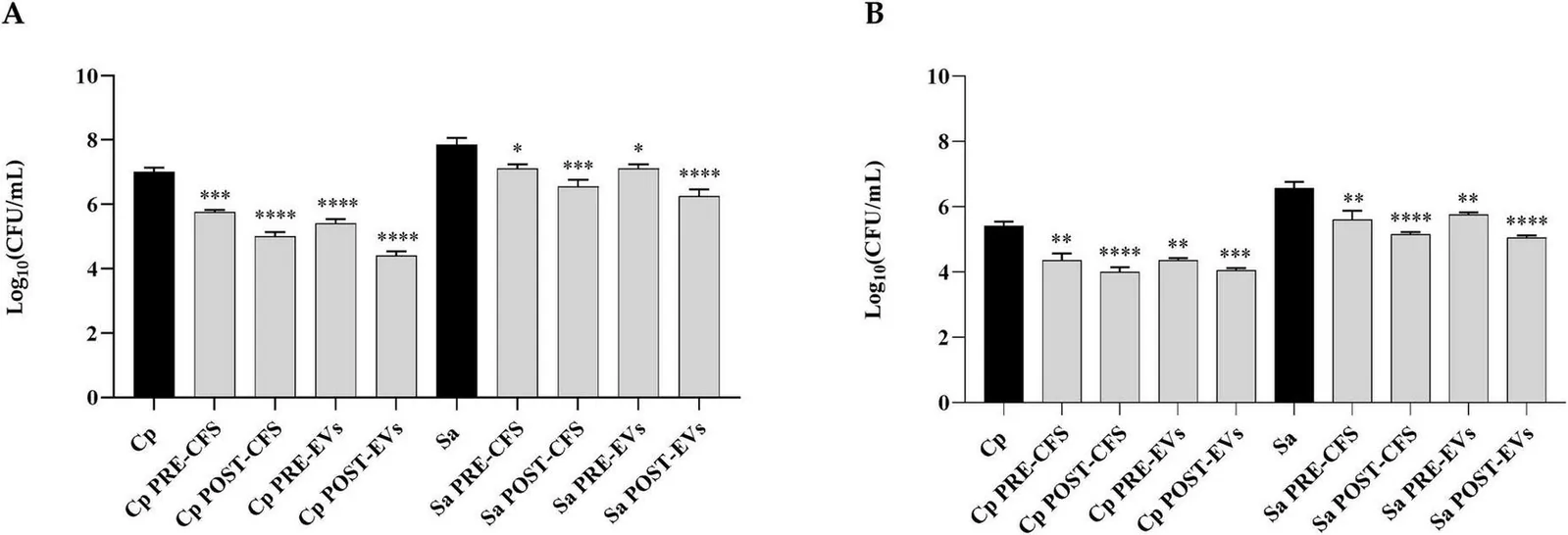
Effect of CFS and EVs on the adhesion and invasion of *C. parapsilosis* (Cp) and *S. aureus* (Sa) in HaCaT cells. **(A)** Adhesion of *C. parapsilosis* and *S. aureus* to HaCaT cells after pre- or post-treatment with CFS (500 μg/mL) or EVs (100 μg/mL). **(B)** Invasion of *C. parapsilosis* and *S. aureus* into HaCaT cells under the same experimental conditions. Data are expressed as Log_10_ (CFU/mL) and presented as mean ± SD from three independent biological experiments. Statistical significance was calculated versus the corresponding untreated infected control using two-way ANOVA followed by Tukey’s multiple-comparison test (*p = 0.05, **p = 0.01, ***p = 0.001, ****p = 0.0001).

An *in vitro* adhesion assay using HaCaT cells was performed to evaluate the adhesive properties of the two isolates, in order to gain a better understanding of the role of CFS and EVs in inhibiting adhesion and invasion to host surfaces. The results are shown in Figure 4. In Figure 4A, both pre-treatment and post-treatment decreased microbial adhesion compared with untreated infected controls for both strains. C. parapsilosis adhesion decreased from approximately 6.9 log_10_ CFU/mL in the untreated control to 5.6 and 5.0 log_10_ CFU/mL after CFS pre- and post-treatment, respectively. EVs further reduced adhesion to about 5.2 log_10_ CFU/mL in pre-treatment and 4.4 log_10_ CFU/mL in post-treatment. A similar but less pronounced effect was observed for *S. aureus*, whose adhesion decreased from approximately 7.8 log_10_ CFU/mL in the untreated control to 6.8 and 6.6 log_10_ CFU/mL after CFS pre- and post-treatment, respectively, and to 7.0 and 6.1 log_10_ CFU/mL after EV pre- and post-treatment. Overall, the inhibitory effect was more evident in *C. parapsilosis* than in *S. aureus*, and post-treatment generally appeared more effective than pre-treatment. Among the tested conditions, EVs, particularly in post-treatment, showed the strongest reduction in adhesion. Similar trend was observed for microbial invasion (Figure 4B). Both CFS and EVs significantly lowered the number of invading cells in HaCaT monolayers infected with either *C. parapsilosis* or *S. aureus*. In *C. parapsilosis*, invasion decreased from approximately 5.0 log_10_ CFU/mL in untreated infected cells to 4.2 and 3.7 log_10_ CFU/mL after CFS pre- and post-treatment, respectively, and to 4.0 and 3.6 log_10_ CFU/mL after EV pre- and post-treatment. For *S. aureus*, invasion was reduced from approximately 6.4 log_10_ CFU/mL in the untreated control to 5.5 and 5.0 log_10_ CFU/mL following CFS pre- and post-treatment, respectively, and to 5.7 and 4.9 log_10_ CFU/mL following EV pre- and post-treatment. Also in this case, the reduction was more pronounced for *C. parapsilosis*, while *S. aureus* remained less affected, although still significantly reduced compared with the untreated control.

### 3.4 Cytokine production in HaCaT cells under pre- and post-treatment conditions

**FIGURE 5:**
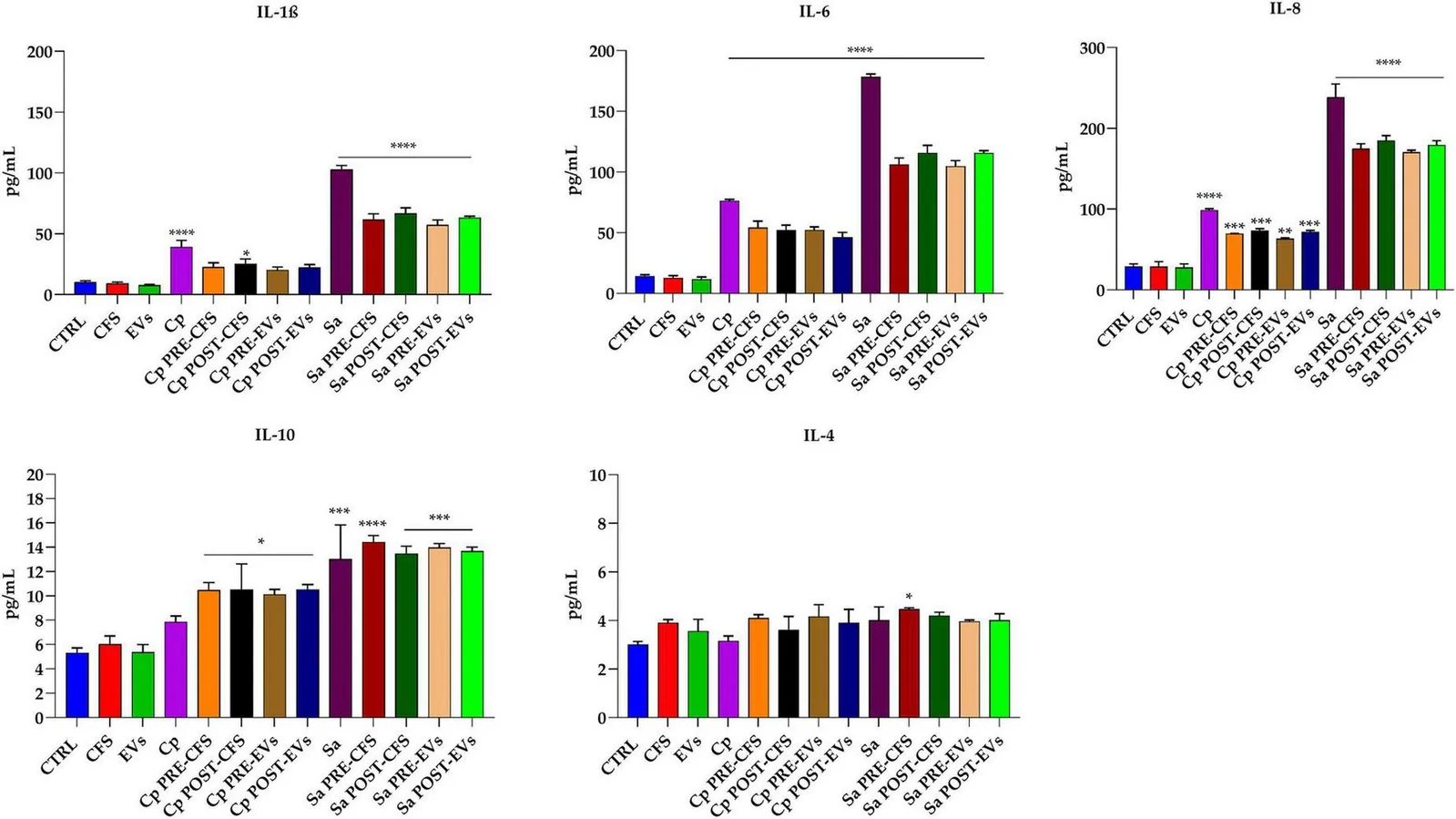
Modulation of cytokine production in HaCaT cells infected with *C. parapsilosis* (Cp) or *S. aureus* (Sa) and pre- or post-treated with CFS (500 μg/mL) or EVs (100 μg/mL). Levels of the pro-inflammatory cytokines IL-1β, IL-6, and IL-8, and the anti-inflammatory cytokines IL-10 and IL-4, were measured in cell supernatants. Data are expressed as pg/mL and presented as mean ± SD from three independent biological experiments. Statistical significance was calculated versus control using one-way ANOVA followed by Tukey’s multiple-comparison test (*p = 0.05, **p = 0.01, ***p = 0.001, ****p = 0.0001).

The inflammatory response of HaCaT cells induced by both *C. parapsilosis* and *S. aureus* infection, as well as by CFS (500 μg/mL) and EVs (100 μg/mL) under pre- and post-treatment conditions was showed in Figure 5. Infection with either microorganism increased the release of the pro-inflammatory cytokines IL-1β, IL-6, and IL-8 compared with untreated controls, with a more pronounced effect observed in *S. aureus*-infected cells than in *C. parapsilosis*-infected cells. For both pathogens, treatment with CFS or EVs, either before or after infection, generally reduced the levels of IL-1β, IL-6, and IL-8 compared with the corresponding infected untreated cells, indicating an attenuation of the pro-inflammatory response. This effect was particularly evident for IL-6 and IL-8, whose levels markedly decreased after both pre- and post-treatment in infected HaCaT cells. In contrast, the anti-inflammatory cytokine IL-10 showed a slight increase in treated cells compared with infected controls. By comparison, IL-4 levels remained substantially unchanged across all experimental conditions, indicating that this cytokine was not significantly affected by either infection or treatment.

### 3.5 *P. kudriavzevii* CFS and EVs promote infected HaCaT cell wound healing

Wound healing is a highly complex and tightly coordinated biological process, and increasing evidence suggests that postbiotic compounds can exert beneficial effects on wound repair (Halper et al., 2003; Karminska et al., 2026; Kuhn et al., 2024; Ong et al., 2020).

**FIGURE 6:**
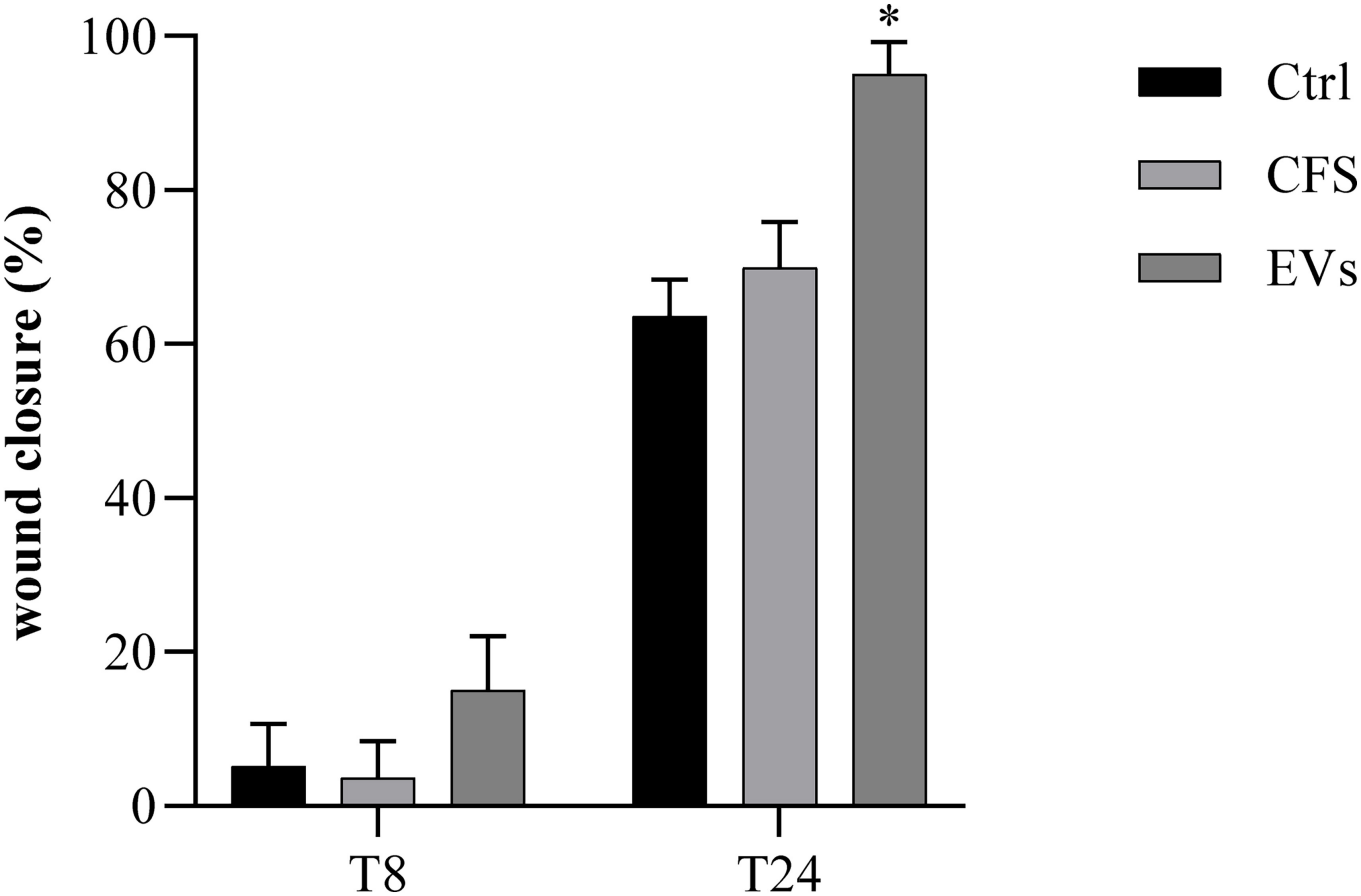
Wound healing activity of *P. kudriavzevii*-derived CFS and EVs in HaCaT cells after 8 and 24 h. Data are expressed as percentage of wound closure compared with T0 and reported as mean ± SD from three independent biological experiments. *p < 0.05 vs untreated control at the same time point using one-way ANOVA followed by Tukey’s multiple-comparison test.

In our study, the impact of CFS and EVs derived from the probiotic *P. kudriavzevii* was investigated using an *in vitro* wound model based on HaCaT cells. Representative images of the wound area at the different time points are reported in Supplementary Figure 1, while the quantitative analysis of wound closure is shown in Figure 6. As shown in Figure 6, treatment with CFS (500 μg/mL) slightly improved wound closure compared with untreated cells, although this increase was not statistically significant. Conversely, EVs (100 μg/mL) markedly enhanced wound closure, reaching almost complete closure, more than 90%, at 24 h and showing a statistically significant increase compared with the untreated control.

**FIGURE 7:**
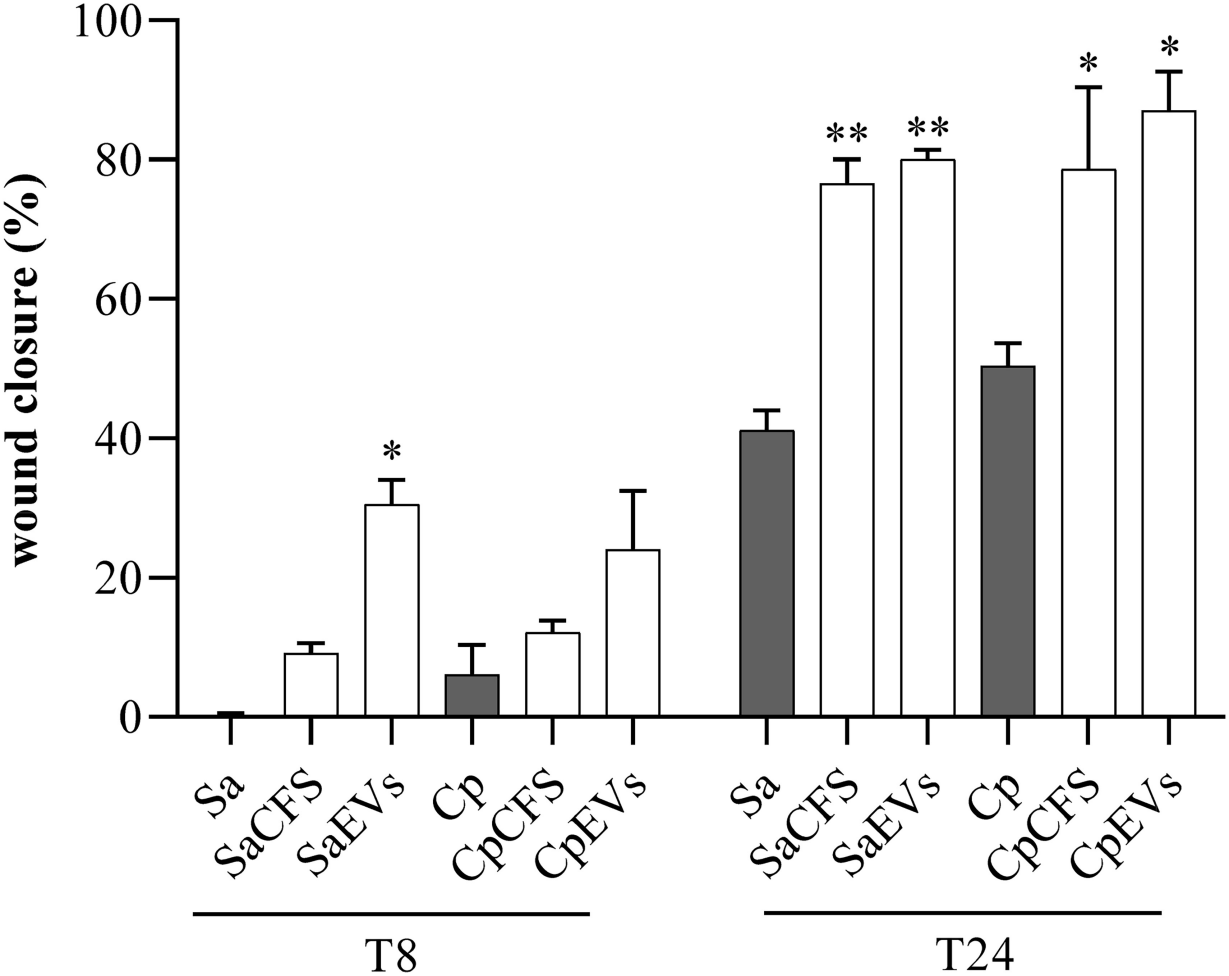
Wound healing activity of *P. kudriavzevii*-derived CFS and EVs in HaCaT cells infected with *S. aureus* (Sa) and *C. parapsilosis* (Cp). Infected wounds were treated with CFS or EVs, and wound closure was quantified after 8 and 24 h. The corresponding non-infected untreated and postbiotic-treated control groups are shown in Figure 6. Data are expressed as percentage of wound closure compared with T0 and reported as mean ± SD from three independent biological experiments. *p < 0.05, **p < 0.01 vs the respective untreated infected control at the same time point (Two-way ANOVA followed by Tukey’s multiple-comparison test).

The effect of the two postbiotics on *in vitro* scratch wound closure was evaluated to investigate their potential application as antimicrobial wound dressings. HaCaT cell monolayers infected with the two clinical isolates were treated with CFS (500 μg/mL) or EVs (100 μg/mL), and images of the scratch area were captured at 8 and 24 h (Supplementary Figure 2). As shown in Figure 7, EVs significantly accelerated scratch closure as early as 8 h in both infection models, with the most pronounced effect observed in the *S. aureus*-infected monolayers. In contrast, CFS produced only a modest increase in scratch closure at this time point, which did not reach statistical significance compared with the corresponding infected controls. After 24 h, both CFS and EVs significantly enhanced scratch closure in the *S. aureus*-infected model, reaching approximately 80% closure. In the *C. parapsilosis*-infected model, EVs remained the most effective treatment, significantly increasing scratch closure compared with untreated infected cells, whereas CFS also promoted scratch closure, although to a lesser extent. Overall, both postbiotic preparations enhanced scratch wound closure under the experimental conditions tested, with EVs consistently exhibiting the strongest effect.

## 4 Discussion

Both bacterial and fungal biofilms play a key role in the development of chronic infections, including those affecting skin, which are often difficult to treat with conventional therapies (Domingue et al., 2020; Fernandes et al., 2020). Atopic dermatitis is a persistent inflammatory skin condition that affects nearly 20% of children worldwide. It presents with marked pruritus, dry skin, and focal inflammatory lesions. The disorder arises from a multifactorial pathophysiology involving the interaction of genetic susceptibility and environmental influences, which together result in disruption of the epidermal barrier and dysregulation of systemic immune responses (Wrzesniewska et al., 2024). Probiotics have been extensively investigated as modulators of the gut microbiota; however, their therapeutic efficacy in atopic dermatitis remains controversial. Among them, *Lactobacillus paracasei* has been widely studied and has demonstrated beneficial effects both *in vitro* and *in vivo*, including the inhibition of pro-inflammatory cytokines and the induction of regulatory T cell–like responses (von der Weid et al., 2001; Weisshaar et al., 2019). In contrast, comparatively little is known about the role of probiotic yeasts. Among these, *Saccharomyces boulardii* has shown anti-inflammatory and immunomodulatory properties, including the modulation of host immune responses and interference with pathogen virulence; however, its role in AD is still poorly characterized and requires further investigation (Pontier-Bres and Czerucka, 2026; Tomičić et al., 2024). The use of live antimicrobial agents for the control of biofilm-associated infections remains limited and is often associated with several problems, including the emergence of pathogen resistance and the potential inactivation or instability of probiotics. In this context, postbiotics have recently emerged as a promising alternative. These molecules have attracted increasing attention due to their stability and multiple beneficial properties, including their ability to support and accelerate the different phases of wound healing (Rawal and Ali, 2023). However, despite growing interest, research on postbiotics is still relatively recent, and their application in AD remains insufficiently explored. In particular*, in vivo* studies in AD patients are still scarce, highlighting the need for further investigation to better define their therapeutic potential. Growing evidence suggests that postbiotics can inhibit biofilm formation, reduce pathogen virulence, and modulate host microenvironment homeostasis. These properties highlight their potential as therapeutic or adjunctive agents in conditions associated with microbial dysbiosis, including atopic dermatitis even if their mechanisms of action and clinical relevance remain to be fully elucidated (Salminen et al., 2021). Among postbiotics, those derived from lactic acid bacteria (LAB), particularly *Lactobacillus* spp., have been the most extensively investigated. Their bioactive components, including metabolites and cell-derived factors, have been shown to interfere with biofilm formation by disrupting microbial communication pathways and modulating extracellular matrix production. The inhibitory effect of LAB postbiotics on early biofilm development appears to be mediated by a complex interplay of multiple metabolites, often resulting in more direct and effective activity compared to live LAB. Furthermore, their favorable safety profile, stability, and bioavailability make them attractive candidates for applications in both clinical and food-related settings. However, despite these promising features and preclinical evidence indicating that LAB-derived postbiotics may reduce contact hypersensitivity responses and the development of atopic skin lesions, their mechanisms of action remain incompletely understood, and further studies are required to optimize their therapeutic application (Liang and Xing, 2023; Moradi et al., 2020; Thorakkattu et al., 2022).

In this work, for the first time, we showed that postbiotics derived from a probiotics yeast *P. kudriavzevii*, isolated in our laboratory from a homemade kefir could offer an alternative approach for complex skin disorders characterized by microbial dysbiosis and persistence of a mixed infection of *C. parapsilosis*/*S. aureus.* Both *C. parapsilosis/S. aureus* play a central role in AD pathophysiology recognized as the most prevalent multi-drug-resistant pathogens often associated with wound infection. They often coexist in multi-species biofilms in chronic wounds, and their association can result in higher virulence and increased tolerance to antimicrobial agents (Gourari-Bouzouina et al., 2026; Pasman et al., 2025). In our previous studies (Maione et al., 2025a; Maione et al., 2026), we successfully isolated and characterized EVs from *P. kudriavzevii* and evaluated their bioactivity using *in vitro* and an *in vivo* assay so as we prepared CFS from the same probiotic candidate. Here, the results reported that the two postbiotics derived from *P. kudriavzevii* inhibited the growth of *C. parapsilosis* and *S. aureus* isolated from chronic skin wound samples of AD patients selected in a preliminary characterization of skin microbiota and mycobiota (Carraturo et al., 2026). Experimental data confirms that both CFS and EVs effectively target biofilm development. Though CFS and EV activities cannot be directly compared in terms of potency because their concentrations were normalized using different metrics, namely dry weight for CFS and total protein content for EVs, they both achieved a significant reduction of biofilm biomass at the respective highest concentrations used, particularly against *S. aureus* biofilms.

The study highlights a hierarchy of susceptibility to postbiotic treatments: *S. aureus* was the most vulnerable, while *C. parapsilosis* exhibited greater tolerance probably due to its complex cell wall. The most significant finding was the higher tolerance of the mixed biofilm compared to mono-species ones. This finding deserves further investigation aimed at clarifying the specific mechanisms involved, and it is aligned with studies reporting that some relationships between *Candida* and bacterial species are synergistic, providing protection to one or both species in the context of dual-species biofilms (Lohse et al., 2018).

The adhesion and invasion assays performed in an *in vitro* human keratinocyte infection model demonstrated that both CFS and EVs significantly reduced the ability of the clinical isolates of *C. parapsilosis* and *S. aureus*, obtained from patients with AD, to interact with host cells. Among the tested preparations, EVs exhibited the greatest inhibitory effect, while *C. parapsilosis* appeared more susceptible than *S. aureus*. These findings suggest that the investigated postbiotics may interfere with microbial colonization and internalization under the experimental conditions tested (Dinić et al., 2024). However, these results should be interpreted within the limitations of the experimental model, which represents a simplified *in vitro* keratinocyte infection system and does not reproduce the complex pathophysiology of AD, including epidermal barrier dysfunction, immune dysregulation, Th2-driven inflammation, and the interactions with the resident skin microbiota. Therefore, the present findings should be considered as a preliminary demonstration of the therapeutic potential of *P. kudriavzevii*-derived postbiotics, warranting further validation in more physiologically relevant experimental models.

Biofilm-forming pathogens play a central role in chronic wound infections by conferring resistance to both antimicrobial treatments and host immune responses, making biofilm disruption a critical target in wound care. While probiotics, particularly *Lactobacillus* spp., have been explored for their ability to modulate skin microbiota and promote healing (Fijan et al., 2019; Lukic et al., 2017), infection-driven inflammation and oxidative stress remain key barriers to effective tissue repair. In this context, postbiotics represent a promising alternative.

Though our experimental approach did not allow to discriminate between cell migration and cell proliferation in the wound healing experiments, our data clearly showed that EVs exerted the most pronounced effect on scratch closure, particularly during the early phase of the assay and under infected conditions. This effect might be related to their ability to deliver bioactive molecules directly to epithelial cells, while also reducing microbial persistence through antibiofilm and anti-adhesive activity.

The inflammatory phase is another crucial process in the wound-healing cascade and prolonged inflammation can lead to pathological conditions that impair the wound-healing process (Deng et al., 2022). During this phase, immune cells, including neutrophils and macrophages, release soluble mediators such as proinflammatory cytokines (IL-1β, IL-6, IL-8) and the anti-inflammatory cytokine (IL-10, IL-4) that orchestrate tissue repair by removing harmful microorganisms and cell debris from damaged tissues. Our results showed that postbiotics treatment affects the cytokine production by attenuating the secretion of IL-6 and IL-8 in response to a pro-inflammatory signal. Consistent with these findings, recent studies with postbiotics have also demonstrated their anti-inflammatory effects. For instance, *L. plantarum* as whole cells downregulated the transcription of TNF-α and IL-8 in LPS stimulated macrophages (Rocchetti et al., 2024).

On the whole, the results of this study highlights the potential of postbiotics derived from a probiotic yeast in the management of chronic skin lesions associated with AD. However, further investigation is needed to develop suitable formulations. In this context, controlled drug delivery systems may play a crucial role in enhancing wound healing outcomes. Hydrogel-based biocomposites, for example (Sadeghi et al., 2026; Zhang et al., 2024), have been shown to improve drug release kinetics, increase therapeutic bioavailability, and reduce dosing frequency.

## 5 Conclusion

Although this study was conducted on a limited number of clinical isolates and relied primarily on simplified *in vitro* models and phenotypic assays, it provides the first evidence that *P. kudriavzevii*-derived postbiotics exert multiple beneficial biological activities against skin pathogens. Both postbiotic preparations inhibited microbial growth in a dose- and time-dependent manner, reduced biofilm formation, impaired microbial adhesion and invasion of human keratinocytes, modulated inflammatory responses, and promoted wound healing. Nevertheless, the molecular mechanisms underlying these effects, as well as the specific bioactive compounds responsible for the observed activities, remain to be elucidated. Furthermore, biofilm assessment was mainly based on crystal violet staining, which quantifies total biomass but does not provide information on microbial viability or biofilm architecture. Therefore, future studies integrating metabolomic, transcriptomic, and proteomic analyses, together with advanced imaging techniques, larger collections of clinical isolates, and more physiologically relevant *in vitro* and *in vivo* models, will be required to validate these findings and clarify their translational potential. Overall, our results highlight *P. kudriavzevii*-derived postbiotics as promising candidates for the development of innovative therapeutic strategies for chronic wound infections and biofilm-associated skin diseases.

## Data Availability

The datasets presented in this study can be found in online repositories. The names of the repository/repositories and accession number(s) can be found in the article/Supplementary material.
