# Supplementary material for: Targeting polymicrobial skin infections in atopic dermatitis: role of Pichia kudriavzevii postbiotics as innovative tools for human health
Source: Front Microbiol. 2026 Aug 6;17:1919460. doi: 10.3389/fmicb.2026.1919460 (PMC13490058; doi:10.3389/fmicb.2026.1919460)
Supplement: Supplementary file 1 [file Table_1.docx]

Supplementary Material

Supplementary Table 1. Effect of DMSO vehicle controls on the experimental parameters evaluated. Data are expressed as mean ± SD. DMSO vehicle controls were compared with the corresponding untreated controls. ns, not significant (t-test).

| **Assay** | **Experimental condition** | **Untreated control** | **DMSO vehicle control** | **Final DMSO concentration** | **Statistical outcome** |
| --- | --- | --- | --- | --- | --- |
| MIC / microbial growth | *C. parapsilosis* | 0.82 ± 0.05 OD590 | 0.80 ± 0.06 OD590 | 1.0% | ns |
| MIC / microbial growth | *S. aureus* | 0.91 ± 0.07 OD590 | 0.89 ± 0.05 OD590 | 1.0% | ns |
| Biofilm formation | *C. parapsilosis* | 0.89 ± 0.04 OD570 | 0.87 ± 0.05 OD570 | 0.5% | ns |
| Biofilm formation | *S. aureus* | 0.75 ± 0.06 OD570 | 0.73 ± 0.05 OD570 | 0.5% | ns |
| Biofilm formation | Mixed biofilm | 2.26 ± 0.12 OD570 | 2.21 ± 0.14 OD570 | 0.5% | ns |
| Adhesion | *C. parapsilosis* | 6.90 ± 0.18 log10 CFU/mL | 6.84 ± 0.20 log10 CFU/mL | 0.5% | ns |
| Adhesion | *S. aureus* | 7.85 ± 0.16 log10 CFU/mL | 7.74 ± 0.19 log10 CFU/mL | 0.5% | ns |
| Invasion | *C. parapsilosis* | 5.40 ± 0.14 log10 CFU/mL | 4.96 ± 0.16 log10 CFU/mL | 0.5% | ns |
| Invasion | *S. aureus* | 6.55 ± 0.17 log10 CFU/mL | 6.34 ± 0.15 log10 CFU/mL | 0.5% | ns |
| Wound healing, 24 h | HaCaT | 63.1 ± 4.2% | 66.8 ± 4.5% | 0.5% | ns |
| Cytokine assay | HaCaT | Parameter-dependent | Comparable to untreated control | 0.5% | ns |

**Supplementary Figure 1.** Representative images of the scratch wound healing assay in HaCaT cells treated with *P. kudriavzevii*-derived CFS and EVs. Images were captured at T0, T8, and T24 using an inverted phase-contrast microscope at 4× magnification. Untreated cells were used as control (CTRL).

**Supplementary Figure 2.** Wound healing activity of *P. kudriavzevii*-derived CFS and EVs in infected HaCaT cells. Representative phase-contrast microscopy images show wound closure progression in HaCaT monolayers infected with *S. aureus* (Sa) or *C. parapsilosis* (Cp) and treated with CFS or EVs. Images were acquired at T0, T8, and T24 using an inverted phase-contrast microscope at 4× magnification.
